# Child temperament predicts the adiposity rebound. A 9-year prospective sibling control study

**DOI:** 10.1371/journal.pone.0207279

**Published:** 2018-11-09

**Authors:** Margarete E. Vollrath, Sarah E. Hampson, Sandrine Péneau, Marie Françoise Rolland-Cachera, Eivind Ystrom

**Affiliations:** 1 Division of Mental and Physical Health, Norwegian Institute of Public Health, Oslo, Norway; 2 Department of Psychology, Faculty of the Social Sciences, University of Oslo, Oslo, Norway; 3 Oregon Research Institute, Eugene, OR, United States of America; 4 Nutritional Epidemiology Research Team (EREN), Centre of Research in Epidemiology and Statistics Sorbonne Paris Cité (CRESS), Paris 13 University, Inserm (U1153), Inra (U1125), Cnam, COMUE Sorbonne Paris Cité, Bobigny, France; 5 PharmacoEpidemiology and Drug Safety Research Group, School of Pharmacy, Faculty of Mathematics and Natural Sciences, University of Oslo, Oslo, Norway; University Complutense of Madrid, SPAIN

## Abstract

The goal of this study is to examine the extent to which child temperament predicts the adiposity rebound, a steep increase in the body mass index (BMI) between ages 5 and 7 years. If this increase occurs at an earlier age, the risk for later obesity is elevated. To improve the accuracy of the examination, we use a genetically informed design, a sibling-control study, to control for genetic and familial confounding. We hypothesize that temperament traits tapping negative emotionality, approach and avoidance are associated with the adiposity rebound.

**Methods:**

We repeatedly examined 25889 siblings within the Norwegian Mother and Child Cohort Study, following them from the mothers’ pregnancy through child age 8 years. Information on the children’s height and weight was collected by means of health registries and maternal reports. Information on the siblings’ temperament was collected by questionnaires completed when they were 1.5, 3, and 5 years old. We examined the associations of temperament at different child ages with the timing of the adiposity rebound among siblings and controls by means of growth curve and multilevel analyses.

**Results:**

Within siblings, high scores on the approach trait of sociability predicted an earlier adiposity rebound and high scores on the avoidance trait of shyness predicted a later adiposity rebound with timing differences ranging between 6 and 16 weeks. Surprisingly, negative emotionality did not predict the adiposity rebound. The associations between temperament and the adiposity rebound increased with increasing child age. The results within controls—comparing siblings with the population, broadly paralleled those within siblings,

**Conclusions:**

The findings encourage the notion that child temperament functions as an early marker for the adiposity rebound. Future studies may advance our knowledge by including measures of child personality along the taxonomy of the adult Five Personality Factors.

## Introduction

Obesity in adulthood is a major risk factor for chronic diseases and death worldwide [1], and being obese in childhood has been regarded as an important stepping stone on this path. Yet, obesity during the earlier years of childhood is a poor predictor of adult obesity, with low relative risk rates. Moreover, 70% of obese adults were not obese in childhood [2, 3]. A better predictor than a specific weight status in childhood, is the BMI-for-age curve. This curve shows ascending and descending phases, reflecting stages of body fat development in children. After infancy, this curve falls, then starts to rise again. The age corresponding to the lowest point of that curve is the Adiposity Rebound [4, 5]. Usually, the adiposity rebound occurs between the age of 5 to 7 years, and the earlier it occurs, the higher is the risk for adult obesity [4–7].

Risk factors for an early timing of the adiposity rebound are the fetal environment [8], parental feeding strategies [9], and early nutrition and diet [10, 11]. Another risk factor may be temperament, i.e. children’s biologically-based differences in reactivity and self-regulation [12]. Temperament traits comprise negative emotionality, activity, extraversion, regularity or restraint, and shyness or inhibition [12, 13]. Negative emotionality involves high reactivity to stress expressed as fearfulness or anger. Activity involves motor activity as well as restlessness. Extraversion (or surgency, sociability) manifests itself as seeking out and enjoying social activities and relations. Restraint and regularity are characterized by perseverance and low impulsiveness. Shyness reflects fear and embarrassment vis-a-vis strangers.

At present, the extent of the association of children’s temperament with the adiposity rebound is still unknown. To generate hypotheses about potential associations, we consulted the literature on the associations between child temperament on the one hand and children’s weight, diet and eating on the other hand. Regarding weight status and early growth, associations with temperament have been established. The trait of negative emotionality is a key predictor of overweight in children and adolescents [14–17]. Self-regulation, low impulsiveness and restraint appear to protect from obesity [14, 15, 17–20]. Temperamental activity has shown an association with lower body weight in children [21–23]. Extraversion (or its subtraits surgency and sociability) is a risk factor for rapid growth and later obesity [15, 20, 24–26]. Some studies also showed positive relations between social anxiety, a construct similar to shyness, and higher body weight [27–29].

Temperament is also related to children’s appetite [30, 31], food enjoyment [32], satiety responsiveness, external eating [33–37], and preferences for sweet food and drinks [19, 38]. Several mechanisms may underlie these relations. Savory, palatable foods and eating in the absence of hunger serve as means to dampen negative emotions such as anxiety, depression, and anger, and this may explain why greater consumption of sweet foods and external eating are related to child negative emotionality [39–45]. Restraint and self-control are related to self-regulation of diet and eating [34, 45–49]. Food has strong intrinsic reward and punishment potentials [50], whereby savory foods trigger approach motivations, and unpleasant foods engender avoidance motivations. This mechanism may explain why extraverted children, having higher sensitivity to rewards [51], show greater enjoyment of food, and preference for palatable foods [47, 48]. Shy, anxious children, in contrast, show food neophobia, i.e. fear of tasting new foods, and are slow, fussy eaters [33, 36, 52].

A general problem when studying the association of any predictor with weight and growth is confounding, the presence of common causes for the exposure and the outcome. In behavioural genetics, one distinguishes between genetic confounding and environmental confounding. Genetic confounding refers to the heritability of the outcome and the predictors. The BMI is highly heritable [53], with estimates ranging from 50% to 70%, and sibling correlations over *r* = 0.86 [54]. Weight for height growth rates during childhood are heritable as well [55]. High heritability is also found for temperament or personality, with an average estimate of 47% across 1 774 twin studies [56]. Heritability also plays a role for potential mediators of the association between temperament and BMI, for instance caloric intake, appetite, and physical activity [57, 58]. Environmental confounding refers to wide array of circumstances during childhood creating an obesogenic environment, such as socio-economic status, parental feeding styles, health-related attitudes, nutrition, as well as the family’s activity habits [59–63].

Twin studies are the best method to parse genetic, shared environmental and unique environmental effects and thus control for confounding. However, suitable twin samples are rare. Sibling studies offer a partial solution to confounding, as they control for genetic and shared environmental effects. This is because siblings share 50% of their genes, and 100% of their familial environment [64]. For example, siblings live in the same household, in the same neighbourhood, share the family’s meals, and partake in family activities, while also attending the same kindergarten and school. In adults, a sibling control study for instance showed that the family environment attenuated the effects of conscientiousness on overweight [65].

The goal of the present study is to examine the associations of children’s temperament with the adiposity rebound. We will use a prospective sibling-control study featuring three assessments of temperament between the ages of 18 months and five years, and seven assessments of weight between birth and age eight years. By investigating the effects of temperament at three time points, we will be able to determine how early this marker can foretell the adiposity rebound. The temperamental traits considered in this study are negative emotionality, activity, sociability, and shyness. We expect, based on the outlined literature, that children scoring high in negative emotionality and sociability will be more likely to have an earlier adiposity rebound, translating into a higher risk for later obesity. More tentatively, we expect that children scoring high in activity and shyness have a later adiposity rebound, translating into a lower risk for later obesity.

## Material and methods

### Design and participants

Siblings were culled from The Norwegian Mother and Child Study (MoBa), an ongoing prospective study with repeated assessments of mothers and children starting in pregnancy. This study is conducted by the Norwegian Institute of Public Health, for details see [66]. In brief, between 1999 and 2008, pregnant women in the catchment area of 50 hospitals and maternity units across Norway received a postal invitation to attend their first free ultrasound scan scheduled between week 17 and 18 of the pregnancy. This invitation also included a letter inviting the pregnant women to participate in the MoBa study. The letter also contained a consent form, the first MoBa questionnaire, and an information brochure. In all, 44% of the invited women participated and provided written consent. Today, the MoBa study includes 95 200 mothers, and their 114 247 living children. Among these, there are 25 889 living biological siblings born to 12 550 mothers, 48.5% girls and 51.5% boys. To assure that all siblings resulted from different pregnancies, we excluded co-twins, co-triplets, and co-quadruplets and included only the first-borns.

MoBa has all necessary concessions from the Norwegian Data Protection Authority and ethical clearance from the responsible ethical committees across the counties in Norway [66]. This specific article received ethical clearance from the Regional Ethics Committee in South East Norway (Ref. 2016/398).

### Assessments

To assess the children’s *temperament*, mothers completed a validated 12-item short form of the Emotionality Activity and Sociability (EAS) temperament survey for children [67], when their children were ages 1.5, 3 and 5 years old. This questionnaire yields four scales: 1. Negative emotionality, characterized by unregulated negative emotions; 2. Activity, a proxy for involuntary activity in children; 3. Sociability, the tendency to enjoy spending time with others rather than alone; 4. Shyness, the tendency to feel distressed by and avoid unfamiliar persons and novel situations. Each item is scored on 5-point response categories (from untypical to very typical). Valid scores were available for 92.5%, 76.5%, and 53.7% of the sample at 1.5, 3, and 5 years, respectively. Unfortunately, the EAS does not include a self-regulation or restraint scale.

Information on the child’s weight and length was available for seven time−points between the child’s birth and the child’s 8^th^ birthday. At birth, this data was obtained from the Medical Birth Registry of Norway [66, 68]. Through age 4 years, mothers reported from records of objective measurements provided to them by national health-stations for children. For the assessments at 5 years, 7 years, and 8 years, mothers self-reported the children’s height and weight. The children’s BMI was calculated as kg/m^2^. Valid measures of BMI were available for 100%, 79.5%, 49.0%, 61.4%, 47.5%, 55.7%, and 36.5% of the sample at 0, 1.5, 2, 3, 5, 7, and 8 years, respectively.

Socio-demographic familial confounders such as parents’ socio-economic status, education, place of residence, and the familial dietary and activity environment are constant when comparing siblings. The same is true for genetic confounders such as the parents’ BMI, health, and temperament. However, individual confounders need to be adjusted for, so all analyses were controlled for the child’s gender, gestational age, standardized weight and height at birth, and the mothers’ parity.

### Statistical analysis

The sibling control analysis compares siblings with each other (“within siblings”) and siblings with other children in the same sample (“sibling-controls” or “within population”). The outcome is the expected change in adiposity rebound when a child differs in temperament compared to his or her siblings. By comparing siblings within the same family, 100% of the familial environment (e.g. the parents’ SES) and 50% of the siblings’ genes are adjusted for by design.

For multiple pregnancies (twins, triplets), only the first-born from each pregnancy was included in the analyses. Within siblings, temperament was centered on the average temperament score of the siblings of the same mother (mean temperament score of siblings minus individual temperament score of each sibling). Thus, for example, a positive score means that a specific sibling has a higher score than the average score of the siblings within that family. In the sibling-control analysis, individual scores are compared to those of other children in that family. Subsequently, each child’s temperament score was kept as a continuous variable, but scaled along ± 2 standard deviations.

The timing of the children’s adiposity rebound was estimated based on their individual BMI trajectories using a quadratic curve (parabola) where the lowest point (vertex) corresponds to the adiposity rebound. The mathematical characteristics of these trajectories (i.e. their slopes and intercept) were allowed to vary across children rendering three random latent variables: an intercept, a linear growth effect, and a quadratic growth effect. We regressed the random effects of the growth curve on the temperament categories and the covariates. Dependency between siblings was accounted for by using a standard sandwich estimator in Mplus 7.1; a method rendering correct standard errors. We estimated all models using full information maximum likelihood (FIML). FIML allows estimating data missing due to attrition or other forms of non-response, hence we could include all children with valid BMI data on one or more occasions. This means that our analyses based on the *missing at random assumption* and accounts for all missing data covarying with observed covariates. An example would be that mothers of obese children drop out of the study.

## Results

We estimated the mean adiposity rebound to occur at age 64.3 months (standard error = 0.02), corresponding to age 5 years, 4 months and 1 week. The average BMI at the adiposity rebound was 15.6. Furthermore, the adiposity rebound of boys occurred on average 16 weeks earlier than the adiposity rebound in girls.

Table 1 shows that the mean gestational age of the siblings was 277.57 days, which is 2.5 days longer than the 275.1 days than the average gestational age of all children born in Norway between the years 2001 and 2008 (Table 1). Table 1 also shows a stable BMI development across 18 months through 3 years, with a slight drop between 5 and 7 years.

**Table 1:** Child birthweight, gestational age, and BMIs at different ages.

	Mean	Standard deviation
Gestational age (days)	277.57	14.41
Birth weight (g)	3532.51	624.01
BMI 18 months	16.74	1.34
BMI 2 years	16.48	1.41
BMI 3 years	16.13	1.48
BMI 5 years	15.57	1.59
BMI 7 years	15.80	1.85
BMI 8 years	16.24	2.02
Maternal age at birth (years)	30.25	4.11

BMI = Body Mass Index

The correlations between the temperamental scales of the EAS across all three measurements, adjusted for age, are presented in Table 2, with controls (the entire sample) above the diagonal, and siblings (adjusted for age and dependency) below the diagonal. The correlations for controls and siblings were very similar, with siblings showing slightly lower correlations. The correlations between activity and sociability were very high, suggesting a common core of both traits. Emotionality showed small correlations with the three other temperament traits. There were negative correlations of approximately equal size between shyness and sociability and shyness and activity.

**Table 2:** Age-adjusted correlations between EAS temperament scales among controls and within siblings.

Controls	Siblings			
	Emotionality	Activity	Sociability	Shyness
Emotionality	—	0.07	0.05	0.12
Activity	0.06	—	0.69	-0.21
Sociability	0.05	0.75	—	-0.24
Shyness	0.16	-0.23	-0.26	—

Note. All correlations are signifikant at p < .001. Siblings above the diagonal, correlations corrected for dependency; controls below the diagonal.

**Fig 1:**
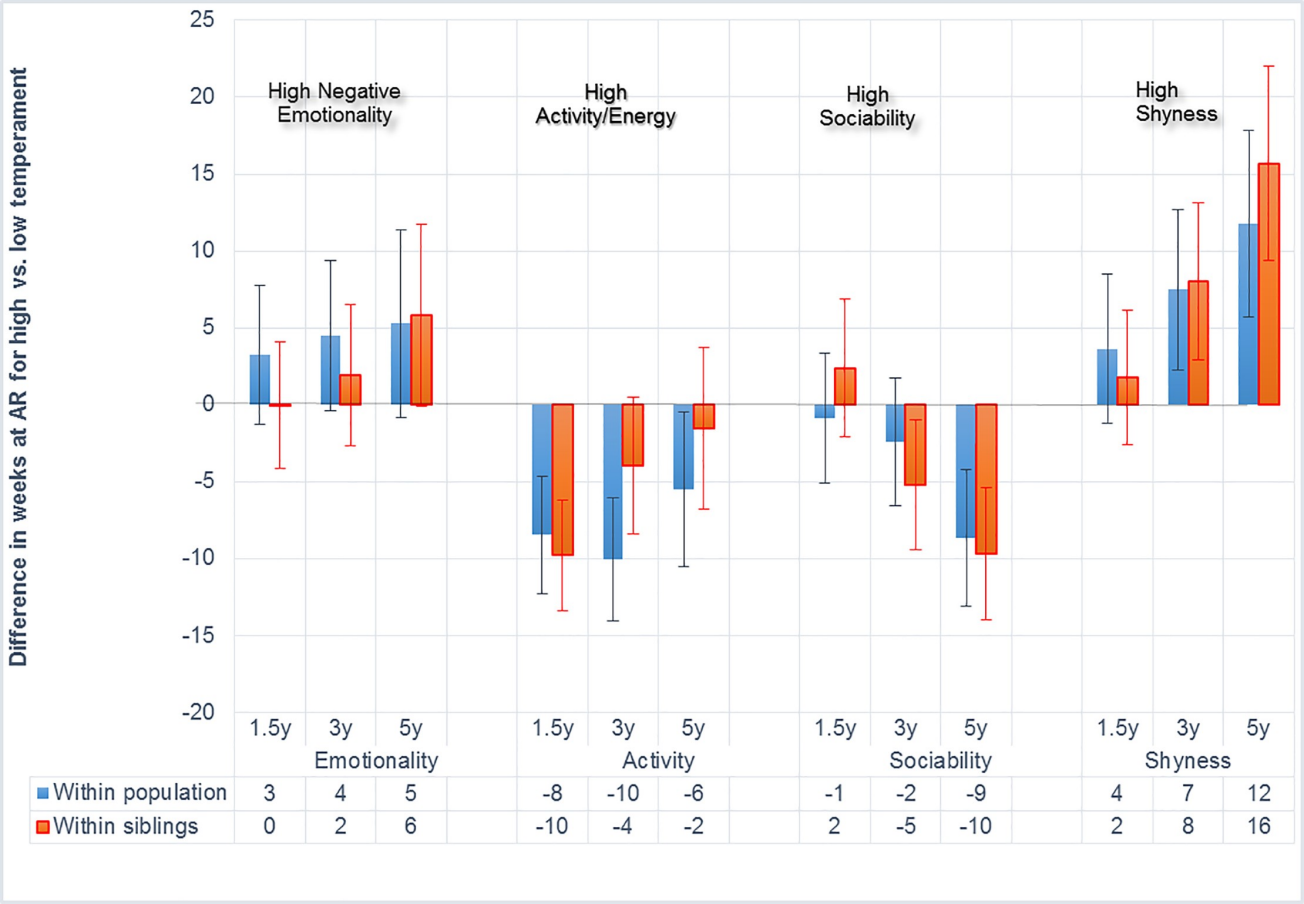
Earlier and later adiposity rebound according to temperament differences. Vertical axis: AR = adiposity rebound differences scaled in weeks. Positive numbers represent later adiposity rebound (lower obesity risk) for those scoring higher than 2 standard deviations on the temperament dimension. Negative numbers represent earlier adiposity rebound (higher obesity risk) for children scoring higher than 2 standard deviations. Red columns: within siblings = comparison of the high scoring siblings’timing of the adiposity rebound with that of the other sibling. Blue columns: within population = comparison of the high scoring siblings’ timing of the adiposity rebound to that of controls (unrelated children). Numbers in the data table represent weeks. Vertical bars represent the width of the 95% confidence interval. Confidence intervals staying within one end of the bars indicate significance *p* ≤ 0.05. y = age in years at temperament assessment.

Fig 1 contains three types of information: 1. it shows differences of the timing of the adiposity rebound in weeks for children scoring lower or higher on each temperament trait (along ±2 standard deviations). 2. It shows differences in the timing of the adiposity rebound for each trait a three time points. 3. It shows differences in the timing of the adiposity rebound within siblings and within the population.

Negative emotionality, the trait capturing children’s moodiness and irritability, was not associated with the adiposity rebound at any age, neither within siblings nor within the population. Activity, the trait indicating children’s energy and vigor, was associated with an earlier adiposity rebound at 1.5 years. Sociability, the trait indicating pleasure, gregariousness and reward orientation, was not associated with the adiposity rebound within the population at 1.5 and 3 years. Within siblings, however, sociability was associated with a 5 weeks *earlier* adiposity rebound at 3 years and a 10 weeks *earlier* rebound at 5 years. Shyness, the trait capturing fear of strangers and novelty, as well as avoidance of social contacts, was associated with a *later* adiposity rebound both within the population and within siblings at child ages 3 and 5 years. Within siblings, the timing differences were 8 and 16 weeks, respectively.

### Discussion and conclusion

The present study adds new information to the child obesity literature by examining the association of early childhood temperament with the timing of the adiposity rebound. Sociability, a temperament trait representing approach motivation was associated with an earlier adiposity rebound, i.e. a higher risk for later obesity. In contrast, shyness, a temperament trait representing social anxiety, avoidance and inhibition, was associated with a later adiposity rebound, signifying a lower risk for later obesity. Temperamental activity at 1.5 years was associated with an earlier adiposity rebound, but activity at 3 years and 5 years was no longer significantly associated with the adiposity rebound. Surprisingly, there were no associations between negative emotionality and the adiposity rebound. By using the conservative design of sibling control, these findings are far less confounded by shared genes or shared environment than classical studies.

Contrary to our expectations, negative emotionality was not associated with the adiposity rebound. This null finding was not limited to the siblings in our sample, where lower associations are to be expected. The few and relatively small classical studies examining the relation of the EAS negative emotionality scale with eating yielded contradictory results, showing associations with both picky eating and overeating [69–71]. Activity was not related to the adiposity rebound, except when measured at age 1.5 years. The association of activity with weight is equivocal, this being the result of the wide divergence of assessment methods, ranging from global temperament scales to the highly sophisticated doubly labeled water methods [72]. Sociability showed associations with an earlier adiposity rebound, which is compatible with mechanisms of higher approach motivation, greater sensitivity to rewards and greater enjoyment of food [47, 48, 73]. The remarkable association of shyness with an earlier timing of the adiposity rebound surprised us, because there are few relevant studies with which to compare our findings. However, shyness has been linked to food neophobia—fear of tasting new foods—and picky eating in a few earlier studies [33, 35, 36, 52].

We observed that associations between temperament and the adiposity rebound increased with increasing child age, probably because growing up implies that children assume greater control of all aspects of their diet and eating behaviors.

This study has a strong design, including a very large number of siblings, extensive longitudinal assessments reaching back to the pregnancy and up to the children’s 8^th^ birthday. The homogeneity of the sample with respect to socio-economic status, ethnicity, education, availability of antenatal health care, and other social background factors was an advantage because it reduces variability. There were many assessment points for both temperament and weight, which strengthens the reliability and validity of the assessments. Most importantly, comparing sibling rather than unrelated participants allowed distilling the unique effects of each sibling’s temperament while adjusting for half of the genetic influences and all of the shared family environment.

At the same time, there are limitations. Importantly, the EAS did not assess children’s self-regulation skills and impulse control. Today, temperament research in children is being superseded by the Five Factor personality research, an approach that has been standardized and refined across decades in adults.[74] The five factor personality taxonomy captures aspects of negative emotionality including self-worth, interpersonal behavior, conscientiousness (self-control, dutifulness), extraversion (positive emotions, excitement seeking), and intellect. Conscientiousness is the essential personality trait predicting health-beneficial behavior in adults[75]. With respect to the children’s height and weight, this information was mother-reported from age 4–5 years onwards, resulting in lower reliability and bias in direction of desired weight. Moreover, the data collection was not completed at 8 years, thus needed a higher proportion of imputation. This, in turn inflated the width of the confidence intervals. As a whole, sibling samples are not representative of the entire population, nor are all mothers willing to participate in time-consuming surveys.

In conclusion, our findings underline the importance of temperament in early childhood as risk factor for the timing of the adiposity rebound. Further research regarding children’s self-control and the entire range of the Big Five personality traits in relation to the adiposity rebound is necessary. Factors mediating the association between temperament and age at adiposity fact rebound may help understand the mechanism involved in the risk of obesity and can be useful to improve prevention.
